# Low-Element Image Restoration Based on an Out-of-Order Elimination Algorithm

**DOI:** 10.3390/e21121192

**Published:** 2019-12-04

**Authors:** Yaqin Xie, Jiayin Yu, Xinwu Chen, Qun Ding, Erfu Wang

**Affiliations:** 1 Electronic Engineering College, Heilongjiang University, Harbin 150080, China; 2171299@s.hlju.edu.cn (Y.X.); 2181234@s.hlju.edu.cn (J.Y.); 1984008@hlju.edu.cn (Q.D.); 2 Communications Research Center, Harbin Institute of Technology, Harbin 150001, China; 18b905012@stu.hit.edu.cn

**Keywords:** underdetermined blind-source separation, chaotic system, empirical mode decomposition, FastICA

## Abstract

To reduce the consumption of receiving devices, a number of devices at the receiving end undergo low-element treatment (the number of devices at the receiving end is less than that at the transmitting ends). The underdetermined blind-source separation system is a classic low-element model at the receiving end. Blind signal extraction in an underdetermined system remains an ill-posed problem, as it is difficult to extract all the source signals. To realize fewer devices at the receiving end without information loss, this paper proposes an image restoration method for underdetermined blind-source separation based on an out-of-order elimination algorithm. Firstly, a chaotic system is used to perform hidden transmission of source signals, where the source signals can hardly be observed and confidentiality is guaranteed. Secondly, empirical mode decomposition is used to decompose and complement the missing observed signals, and the fast independent component analysis (FastICA) algorithm is used to obtain part of the source signals. Finally, all the source signals are successfully separated using the out-of-order elimination algorithm and the FastICA algorithm. The results show that the performance of the underdetermined blind separation algorithm is related to the configuration of the transceiver antenna. When the signal is 3 × 4 antenna configuration, the algorithm in this paper is superior to the comparison algorithm in signal recovery, and its separation performance is better for a lower degree of missing array elements. The end result is that the algorithms discussed in this paper can effectively and completely extract all the source signals.

## 1. Introduction

To reduce professional equipment installation for intelligent communication, realize better information transmission with limited resources, and receive a large amount of source information using limited sensors at the receiving end, use of a low-element model at the receiving ends is becoming popular in the field of communication [1,2,3]. Application of low-element treatment for sensors at the receiving ends is highly suitable for areas involving information transmission such as biomedical engineering, seismic monitoring, signal enhancement, and radar and machinery [4,5,6,7,8,9,10]. As the information detected by sensors at each receiving end is the superposition of some source information, independent sources are required to obtain essential information [11]. Therefore, certain auxiliary information processing methods are required to handle the collected information and separate each desired source signal.

Blind-source separation (BSS) involves separating the best estimation of the hidden source signals from certain observed signals (at the receiving end) when the theoretical model of the signal and the source signal are unknown [12,13,14]. Specifically, underdetermined blind-source separation is a low-element model of sensors at the receiving end for signal processing, which remains an ill-posed and abstruse problem for information transmission [15,16,17,18]. At present, there are two types of solutions to address this issue. The first type is based on the statistical properties of source signals, such as uncorrelated autoregressive (AR) model signals [19], nonnegative tensor factorization [20], and beamforming based on a minimum mean square error [21]. The second type is based on the analytical method of signal sparsity, which mainly includes chaotic matrix estimation and source signal restoration methods. In general, a transform-domain sparse representation can yield better source signal estimation [22]. Common time-frequency analytical techniques include Cohen’s class time-frequency distributions [23], short-time Fourier transform (STFT) [24], and empirical mode decomposition [25]. As blind-source separation aims to address the problems of nonlinear signals and the time-frequency analysis uses the non-stationarity of signals, this paper also begins by discussing time-frequency analytical techniques for signal separation.

Currently, although progress has been made in underdetermined blind-source separation based on time-frequency analysis, common methods used still have certain limitations. For example, although STFT can successfully separate the source information with underdetermined blind-source separation, the window function used obscures the time-frequency (TF) representation and is confined by the Heisenberg uncertainty principle, which further restricts the sparsity of a signal [26,27,28]. When a wavelet transform performs underdetermined blind-source separation, various wavelet bases and decomposition layers create a difference between the vortex and approximate signals, thus, influencing the separation of signals and making it difficult to select the wavelet base and decomposition layer number [29,30,31,32,33]. Empirical mode decomposition can cause modal aliasing when dealing with underdetermined blind-source separation, making the separation of source signals difficult [34,35].

Previous transmission carriers mainly consisted of voice information, and information transmission was performed in the forms of text, voice, and images [36]. Generally, images are considered more suitable for modern communication because they are intuitive, capable of carrying a large amount of information, and conducive to observation and summarization [37]. Existing studies on image separation methods based on underdetermined blind-source separation are not sufficient. Studies on low-element receiving ends of images will help address the issue of increasing communication demands. 

To resolve these problems, this paper proposes low-element image restoration based on an out-of-order elimination algorithm. The contributions of this study are as follows: first, chaotic hiding information is used in sensors with low-element receiving ends to protect information at the receiving end. Second, to solve the low-element problem at the receiving end and to prevent modal aliasing, this paper proposes empirical mode decomposition to construct multiple components to increase information through the decomposed simple component signals and suppress modal aliasing using the first intrinsic mode function. Finally, as all the source signals cannot be simultaneously extracted by underdetermined blind-source separation, this paper proposes an out-of-order elimination algorithm to reduce the extracted image information. Next, the mixed information can be used for blind extraction to eliminate repeated blind extractions and ensure complete information extraction.

The rest of the paper is organized as follows: Section 2 introduces the principles of 3D digital chaotic systems and empirical mode decomposition, as well as the theoretical models of underdetermined blind-source separation. Section 3 discusses the development of an underdetermined blind-source separation method based on low-element images and introduces the design scheme and model for constructing multi-component complement system and an out-of-order elimination algorithm using empirical mode decomposition in detail. Section 4 shows the overall flow chart and performance evaluation methods. In Section 5, through simulation, the effect of chaos on information hiding and the approximation effect of blind extraction of source information by the proposed low-element algorithm are analyzed. Section 6 summarizes the algorithms used in this paper.

## 2. Basic Theoretical Method 

### 2.1. Underdeterminate Blind Signal Separation

**Figure 1:**
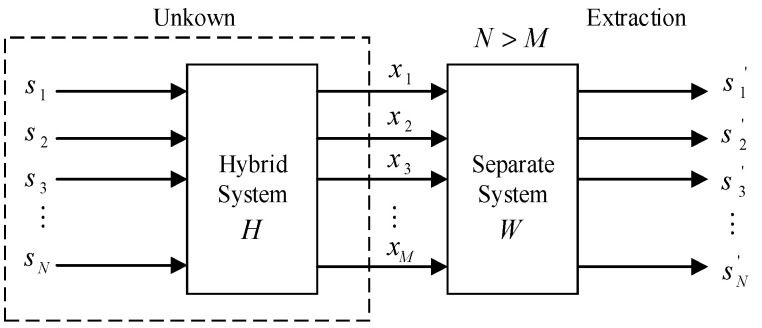
Underdetermined state blind extraction mathematical model.

Underdetermined state blind extraction mathematical model shown in Figure 1. Here “blind” means that the transmitter signal s is unobservable and the characteristics of the hybrid system H are unknown [38,39]. Among them, the independent component analysis (ICA) technology can use the statistical conditions such as independent independence of transmitter signals s statistics to reproduce an unobservable transmitter signal s from the receiving signals x. In the independent component analysis algorithm, fast independent component analysis FastICA is widely used in blind-source separation because of its fast convergence and high stability [40]. Therefore, in the blind-source separation and blind extraction, the FastICA algorithm in independent component analysis will be used in this paper. 

Set $x={\left(x_{1},x_{2},\ldots ,x_{m}\right)}^{T}$ be the m-dimensional zero-mean random receiving signals. It is n linear mixture of an unknown zero mean independent transmitter semaphore $s={\left(s_{1},s_{2},\ldots ,s_{n}\right)}^{T}$. This linear mixed model can be expressed as:

(1) $$x=Hs=\sum_{j=1}^{n}h_{j}s_{j},j=1,2,\ldots ,n$$

Among, $H=[h_{1},h_{2},\ldots ,h_{n}]$ is a *m* × *n*-order full-range transmitter signal mixing matrix; $h_{j}$ is the n-dimensional column vector of the mixing matrix. The Equation (1) is in the form of a matrix:

(2) $$\left[\begin{array}{c} x_{1}(t)\\ \vdots \\ x_{m}(t) \end{array}\right]=\left[\begin{array}{ccc} h_{11} & \cdots & h_{1n}\\ \vdots & & \vdots \\ h_{m1} & \cdots & h_{nm} \end{array}\right]\left[\begin{array}{c} s_{1}(t)\\ \vdots \\ s_{n}(t) \end{array}\right]$$

In the equation: each mixed signal $x_{i}(t)(i=1,\ldots ,m)$ is a random signal, and each receiving $x_{i}(t)$ is a sample of the random signal $x_{i}$ when time is t.

In mixed matrix ***H*** and transmitter signal ***s*** unknown circumstances, only with the sensor detects the mixed receiving signal ***x*** separate transmitter signals is most likely true ***s***, can build a separation matrix $W={\left(w_{ij}\right)}_{n\times n}$. When ***s*** gets the best estimation of ***r*** dimension transmitter signal through the separation matrix W transform, it is output column vector $s^{\prime }={\left(s_{1}^{\prime },s_{2}^{\prime },\ldots ,s_{n}^{\prime }\right)}^{T}$. The solution (or the solution mix model) of this problem can be expressed as:

(3) $$s^{\prime }\left(t)=Wx(t)=WHs(t)=Gs(t\right)$$

In the equation: ***G*** is a global transmission matrix. Under normal circumstances, when ***G = I*** (***I*** is an n×n-order unit matrix), and $s^{\prime }\left(t)=s(t\right)$, thus achieving transmitter signal separation. Where, N is the number of transmitter signals and M is the number of receiving signals. Among them, the number of mixed receiving signals is less than the number of transmitter signals, and an underdetermined mathematical model is obtained.

### 2.2. Chaotic System

**Figure 2:**
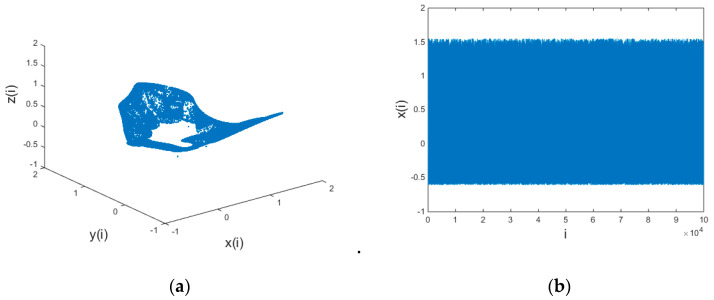
A 3D discrete chaotic schematic: (**a**) three-dimensional view of the chaotic system and (**b**) time domain response graph of x variable of the chaotic system.

Chaotic hiding information ensures secure communication. As low-element underdetermined blind-source separation is problematic, to ensure collection of secured information at the receiving end, this paper uses digital chaos to hide and confidentially transmit the transmitter information [41,42]. The kinetic equation of the chaotic system [43] used in chaotic hiding techniques is shown in (4):

(4) $$\{\begin{array}{l} x\left(i\right)\;=ax(i-1)+by(i-1)+cz(i-1)+dx(i-1)y(i-1)+ex(i-1)z(i-1)+fy(i-1)z(i-1)\\ y\left(i\right)\;=\;x\left(i-1\right)\\ z\left(i\right)\;=\;y\left(i-1\right) \end{array}$$

where a=−0.54,b=−0.25,c=0.79,d=−1.79,e=−1.69,f=−1.78. When the initial values are:

$$\{\begin{array}{l} x\left(0\right)\;=0.63\\ y\left(0\right)\;=0.81\\ z\left(0\right)\;=\;-0.75 \end{array},$$

it can iterate into a chaotic state. The chaotic three-dimensional (3D) diagram and the time-domain response diagram of variable x are shown in Figure 2.

The characteristic analysis of a chaotic system [44] reveals that it has a complex structure and irregular dynamic behavior. It is proven in [43] that a chaotic system possesses a good image encryption effect. Therefore, a 3D hyper-chaotic system works well for chaotic hiding information transmission.

### 2.3. Empirical Mode Decomposition

Empirical Mode Decomposition (Empirical Mode Decomposition—EMD) is the core algorithm of the Hilbert–Huang Transform (HHT) [45]. The EMD algorithm can handle complex and difficult-to-decompose non-stationary signals and decompose them into simple combinations of single-component signals, is a set of better-performing Intrinsic Mode Functions (IMF). The IMF must satisfy the following two conditions [46]:

1. The number of signal zero crossings is equal to or at most equal to the number of extreme points of the IMF;
2. The mean value of the upper envelope constructed by the local minimum value and the local maximum value is zero.

The specific steps of the EMD algorithm [47,48] are: First, find all the extreme points of the original data sequence Y(t), and use the cubic spline curve to fit the upper and lower extreme points, and calculate the average of the upper and lower envelopes. Then, subtract the mean from Y(t) to determine if the difference is IMF, and if not, repeat the above steps until the criterion is satisfied. The $IMF_{j}$ component of signal Y(t) is obtained after decomposition and the remaining terms $r_{n}$:

(5) $$Y(t)=\sum_{j=1}^{n-1}IMF_{j}+r_{n}$$

According to the principle of EMD decomposition, the characteristic scale of the signal can be embodied in each *IMF* component, and the IMF component also represents the internal modal characteristics of the signal. At the same time, it is found that the most complete signal component of the signal Y(t) is the first intrinsic mode function component $IMF_{1}$ after decomposition.

## 3. Building the Algorithm Model

### 3.1. Construct Multi-Component Complementary Method

**Figure 3:**
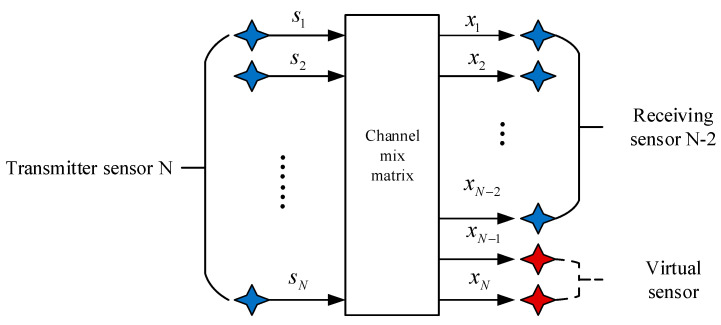
A virtual receiving array model with multicomponent complement method.

The model of the multi-component complementary method is shown in Figure 3. In this model diagram, it is assumed that the number of receiving sensors is less than the number of transmitting sensors. It can be extended to the model that lacks multiple receiving sensors by missing two receiving sensors in this model. In this model, two virtual receiving sensors will be added to make the underdetermined blind-source separation system positive, which is M=N in Figure 1. The supplementary two-way virtual receiving sensor equation is:

(6) $$x_{N-1}(k)=IMF_{1},k=1,2,3,\ldots ,T$$

(7) $$x_{N}(k)={\tilde{x}}_{1}(k)+IM{\tilde{F}}_{1}(k),k=1,2,\ldots ,T$$

(8) $${\tilde{x}}_{1}(k)=\left\{ \begin{array}{l} x_{1}(k),k=2m-1\\ 0,k=2m \end{array}\right.,$$

(9) $$IM{\tilde{F}}_{1}(k)=\left\{ \begin{array}{l} 0,k=2m-1\\ IMF_{1},k=2m \end{array}\right..$$

Firstly, the first virtual signal $x_{N-1}$ of the receiving sensor is constructed, which is the $IMF_{1}$ obtained by the EMD of the receiving end $x_{1}$; Secondly, the virtual signal $x_{N}$ of the second receiving sensor built is obtained by the odd and even combination of $x_{1}$ and $IMF_{1}$. In the underdetermined hybrid system, a new set of received signals can be combined through the above two Equations (6) and (7) to be converted into a positive definite system.

### 3.2. Out-of-Order Elimination Algorithm

**Figure 4:**
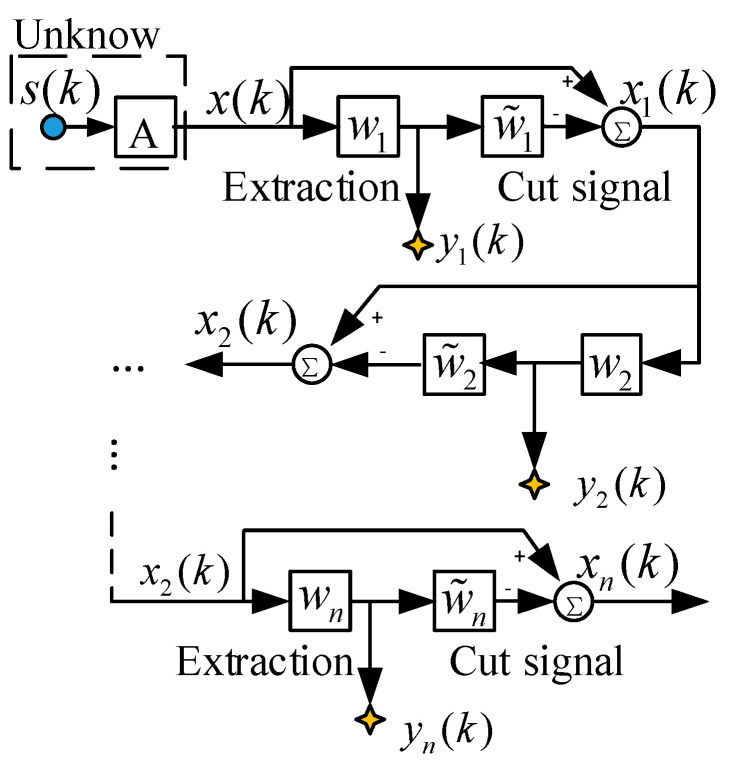
Implementation of extraction and reduction.

The construction of the above multi-component complementary method transforms the underdetermined blind-source separation into a positive definite model that can be extracted blindly. However, considering that only part of the signal may be obtained in one extraction, this paper proposes an algorithm of out-of-order elimination algorithm. The function of this algorithm is to subtract the successfully extracted signal from the mixed received sensor signal and use the remaining mixed signal information for more efficient blind extraction. The model of the algorithm is shown in Figure 4.

The algorithm adopts a hierarchical extraction structure and utilizes two different types of processing modules that are connected in a cascaded manner. The first layer is an extraction module, which is used for blind extraction of mixed signals by linear feature extraction. The second layer is the elimination module, which applies the principle of minimum absolute deviation and subtracts the signal successfully extracted by the first layer from the received signal. 

The j-th extraction module and the elimination module respectively extract the source signal from the input end and subtract the newly extracted source signal. The first subtraction module excludes the newly extracted transmitter signal from the input side mixed signal, and then gives the remaining subtracted mixed signal to the next (j + 1) module for a new round of extraction and elimination.

We will assume that the first source signal $y_{1}(k)\approx s_{i}(k)(i\in \bar{1,n})$ is successfully extracted, and then the successfully extracted information $y_{1}(k)$ is subtracted from the received mixed signal. The process of extracting and subtracting the remaining mixed signals can be used recursively until all the transmitter signals are extracted and terminated when no extraction signal is available. In this process, the following equation can be obtained:

(10) $$x_{j+1}(k)=x_{j}(k)-{\tilde{w}}_{j}y_{j}(k)$$

where ${\tilde{w}}_{j}$ can be optimized by minimizing the cost (energy) function:

(11) $$J_{j}{(\tilde{w}}_{j})=E\{\rho {(x}_{j+1})\}=\frac{1}{2}E\{\sum_{p=1}^{m}x_{j+1,p}^{2}\}$$

where $E\{\rho (x_{j+1})\}$ is the target function, and $y_{j}(k)=w_{j}^{2}x_{j}(k)$. Intuitively, such a target function can be regarded as an energy function. When $y_{j}$ of the extracted source signal is subtracted in the mixed signal of the source, the energy function should reach the minimum value.

## 4. Overall Flowchart and Performance Analysis Indicators

### 4.1. Overall Flow Chart

**Figure 5:**
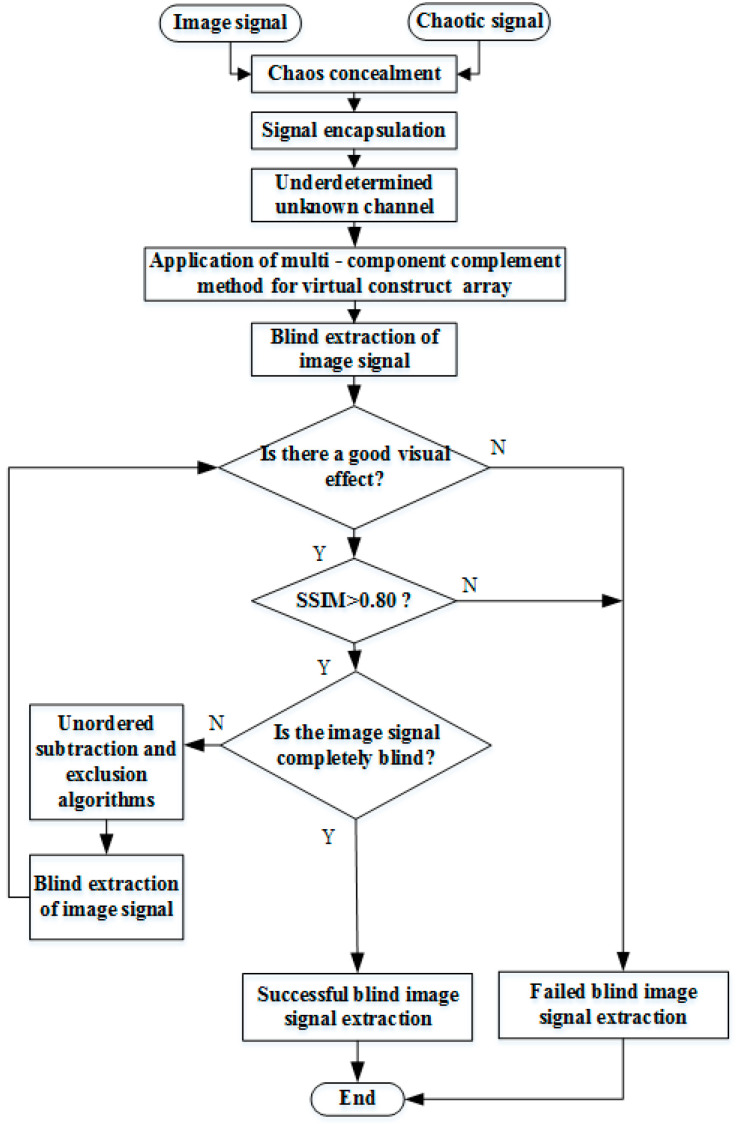
Overall flow chart.

Figure 5 depicts an overall flow chart of low-element image restoration based on the out-of-order elimination algorithm, which describes the complete algorithmic process. First, to ensure that the observed signals at the receiving end are kept confidential, the x-component of the chaotic signal is used as the transmitter signal to transmit alongside other source information. Second, as low-element problems are encountered at the receiving end, the EMD algorithm is used to decompose the virtual signals for constructing multiple components to convert the low-element sensors at the receiving end into a positive definite state, to realize effective blind extraction of the mixed signals. Finally, as it is difficult to simultaneously extract all the transmitter signals of the low-element sensors at the receiving end, the proposed out-of-order elimination algorithm is used to accurately and quickly extract the non-extracted signals.

### 4.2. Performance Analysis Index

In this study, to better see the encryption effect of chaos on digital images, the encrypted image was analyzed according to the information entropy against statistical attack ability [49]. The information entropy describes the information uncertainty. For higher information uncertainty, more information is needed for factual clarification. Therefore, the larger the information entropy of the encrypted image, the more secure the corresponding encryption algorithm. The following equation was used to evaluate the information entropy of the original and encrypted images:

(12) $$H(x)=\overset{2^{n}-1}{\underset{i=0}{\Sigma }}p(x_{i})\log_{2}(p(x_{i}))$$

where $p(x_{i})$ represents the probability at $x_{i}$ and 2^n^ is the number of image pixel values. For a grayscale image, there are 256 gray levels, and the ideal information entropy is close to 8. If the information entropy of an encrypted image is closer to 8, the corresponding encryption method is more secure, and the ability to resist entropy attacks is stronger.

Generally, both qualitative and quantitative performance evaluations of BSSor blind extraction problem-solving algorithms can be performed. As the current study targets blind extraction of image information, a qualitative evaluation can be conducted using subjective visual analysis of the human eye. Primarily, the image information before and after the BSS or blind extraction is compared and analyzed to yield an intuitive discrimination. Quantitative evaluation is performed using performance indicators such as the structural similarity (SSIM) [50], mean-square error (MSE), and peak signal-to-noise ratio (PSNR).

SSIM: From the perspective of image composition, structural information is defined as being independent of brightness and contrast, instead reflecting the properties of the object structure in the scene. The distortion is modeled as a combination of three different factors: brightness, contrast, and structure. The definition is:

(13) $$\mathrm{SSIM}(s,y)=\frac{\left(2\mu_{x}\mu_{y}+c_{1})(2\sigma_{xy}+c_{2}\right)}{\left(\mu_{x}^{2}+\mu_{y}^{2}+c_{1})(\sigma_{x}^{2}+\sigma_{y}^{2}+c_{2}\right)}$$

In the equation, ***x*** is the estimated value of the image information after blind separation or blind extraction, and y is the true value of the image information in the source signal. $\mu_{x},\mu_{y}$ and $\sigma_{x},\sigma_{y}$ are their mean and variance, respectively, $\sigma_{xy}$ is their covariance. $c_{1}={\left(k_{1}L\right)}^{2},c_{2}={\left(k_{2}L\right)}^{2}$ are two constants that are used to maintain stability. L is the dynamic range of the pixel value. $k_{1}=0.01,k_{2}=0.03$. When the two images are exactly the same, the SSIM value is equal to one. In this paper, when the SSIM approaches 1, it can be determined that the blind extracted image has high similarity to the source image information, which is an estimate of the source image information.

MSE: The MSE is defined as:

(14) $$MSE=\frac{1}{M\times N}\sum_{m=0}^{M-1}\sum_{n=0}^{N-1}{\left(s(m,n)-\hat{s}(m,n)\right)}^{2}$$

where, $s,\hat{s}$ represent the images before and after encryption, respectively, and *M* and *N* are the image height and width, respectively. A smaller MSE for an evaluation image restoration indicates higher-accuracy image restoration.

PSNR: The PSNR is essentially identical to the MSE and is defined as:

(15) $$PSNR=10\log_{10}\frac{255^{2}}{MSE}$$

Use of the PSNR is an objective image restoration evaluation method. In general, a higher PSNR value corresponds to less distortion.

In addition, when the global transmission matrix in Equation (3) is the generalized permutation matrix [51], that is, G=PD (P is the permutation matrix, D is the non-singular diagonal scale matrix). The source signal s can be obtained from the signal $s^{\prime }$, which is $s=G^{-1}s^{\prime }$. Then, the estimated performance of G=I is optimal, which means that the global matrix G can also be used as a basis for the separation performance. When the global matrix is the dominant matrix, the separation performance is good. The dominant matrix is that for every row and column in the matrix, only one element approaches 1, while the rest of the elements approach 0.

## 5. Simulation Results and Performance Evaluation

### 5.1. Chaotic Hiding Observation Signals

**Figure 6:**
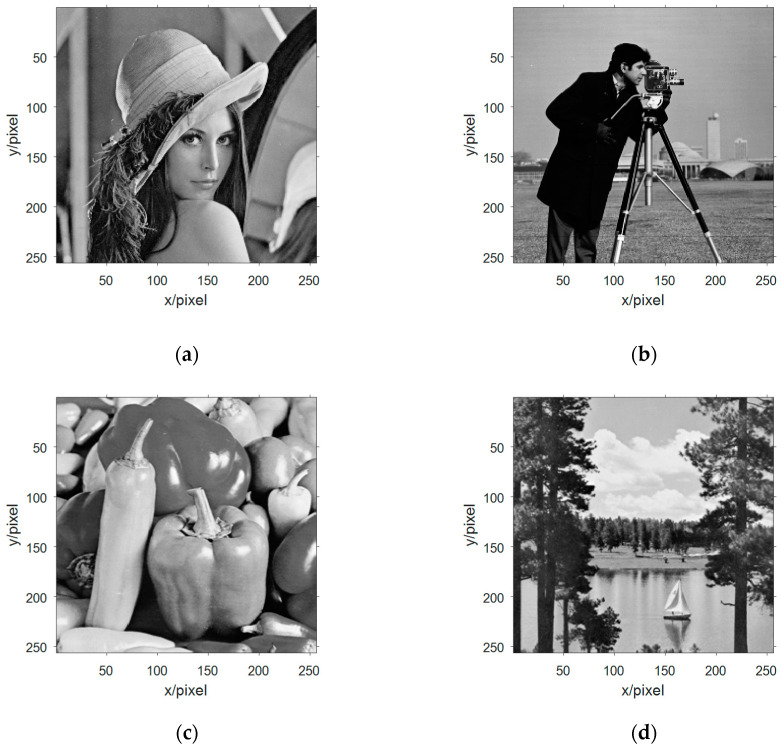
The image information of the source signal: (**a**) image information of the first source; (**b**) image information of the second source; (**c**) image information of the third source; and (**d**) image information of the fourth source.

Selecting the grayscale image information from the four standard test image libraries is shown in Figure 6a–d, and they are converted from a two-dimensional array data into a one-dimensional array of data, and then binarize one-dimensional data. The x-components in the Chen chaotic system are selected and these five signals are packaged as an n-source signal vector.

For the unknown channel of the analog channel, the system randomly generates a 3×5 mixed receiving matrix and the encapsulated data to be aliased to obtain three mixed receiving signals. The randomly generated mixing matrix ***H*** in this system is:

(16) $$H=\left[\begin{array}{ccccc} 0.2971 & 0.1637 & 0.5011 & 0.4800 & 0.5358\\ 0.5160 & 0.2446 & 0.8062 & 0.0803 & 0.9652\\ 0.8847 & 0.3900 & 0.5785 & 0.6677 & 0.2592 \end{array}\right]$$

**Figure 7:**
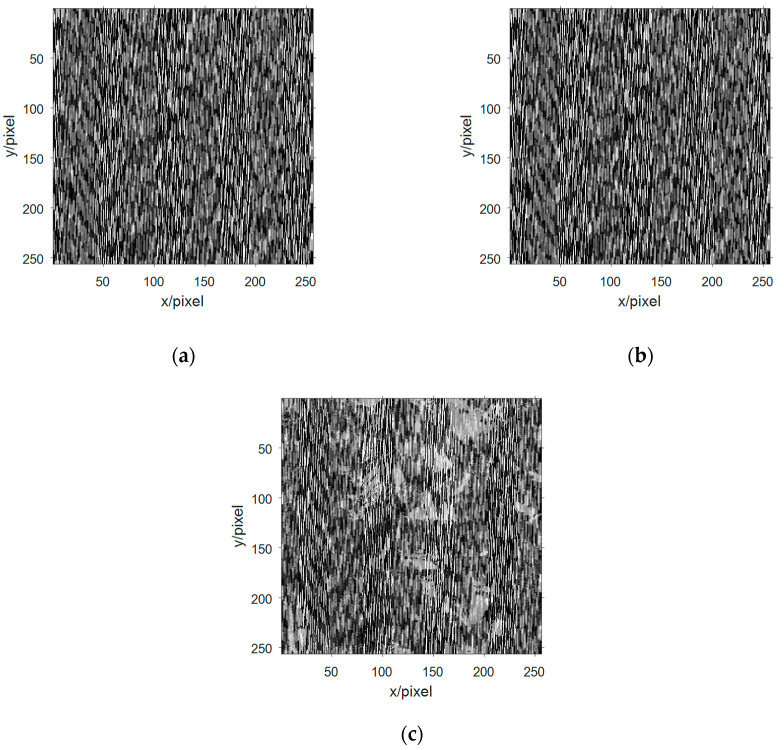
The image information of the observed signal in the multicomponent complement model: (**a**) image information of the first observation signal; (**b**) image information of the second observation signal; and (**c**) image information of the third observation signal.

Decimalizing the data and performing a two-dimensional arraying to obtain three-channel image information as shown in Figure 7a–c. Observing the three-way image information of the mixed receiving signal, the image information is disorderly and disorderly, and the information in the image cannot be recognized. The content of the image information is better obscured by the signals of the 3D chaotic motion system and can no longer be recognized by human eyes.

### 5.2. Security Analysis of the Observed Signals at the Receiving End

#### 5.2.1. Entropy

For a grayscale image, the closer the information entropy of the encrypted image to 8, the more secure the corresponding encryption method, and the stronger the ability to resist entropy attacks. In this study, 3D chaos was selected for entropy analysis of the concealed and encrypted images, as detailed in Table 1.

The four images were encrypted through chaotic masking (four image data plus one chaotic data can be understood as five channels of signal transmission) and underdetermined blind source analysis channels to obtain three-way hiding images. From Table 1, the five signals are encrypted by chaotic hiding and underdetermined blind-source separation channels to obtain three-way encrypted images. Table 1 shows that that when two types of 3D hyper-chaotic systems (Lorenz and 3D chaos) are used for chaotic hiding transmission of information from four source signals (Lena, Cameraman, Lake, and Peppers), the information entropy values of the three mixed receiving signals received by the low-element receiving ends are close to 8. Therefore, the two chaotic signals can provide a good encryption effect and have the ability to resist entropy attacks. The 3D chaotic system is selected for transmission of chaotic hiding information in this study, as the entropy value obtained by it is better than that of Lorenz chaos.

#### 5.2.2. Statistical Analysis 

**Figure 8:**
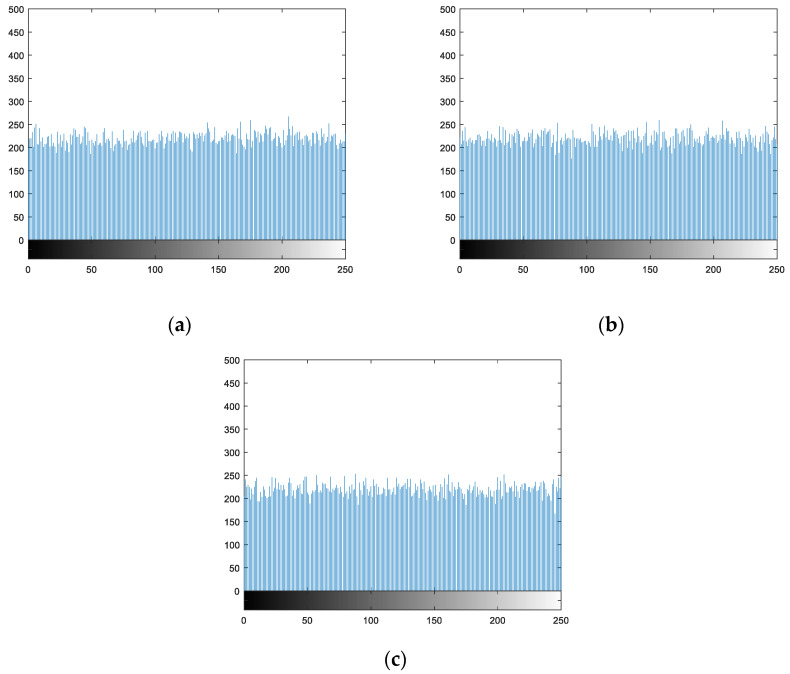
Statistical histograms of hiding images. (**a**)–(**c**) is the statistical histogram of the hiding image in Figure 7.

For Lena, Cameraman, Lake, and Peppers four gray image hiding operations, draw a clear image of the statistics histogram, respectively, and the corresponding hiding statistical histogram of the image. The result is shown in Figure 8, you can see from the picture clear image pixel gray value of occurrence probability is very uneven, but after the hiding image of each pixel gray value probability is very uniform, shows that the hiding image pixel gray value taken the probability of all possible values tend to be equal. Therefore, this algorithm can effectively resist the attack of statistical analysis.

#### 5.2.3. Differential Attack Analysis 

The ability of an image hiding to resist differential attacks can be analyzed with the two indicators: Number of Pixels Change Rate (NPCR) and the Unified Average Changing Intensity (UACI) [52]. The calculation equation for NPCR and UACI are defined as follows:

(17) $$NPCR=\frac{1}{m\times n}\sum_{i=1}^{m}\sum_{j=1}^{n}D\left(i,j\right)\times 100\%$$

(18) $$D\left(i,j\right)=\left\{ \begin{array}{l} 0,C_{1}\left(i,j\right)=C_{2}\left(i,j\right),\\ 1,C_{1}\left(i,j\right)\neq C_{2}\left(i,j\right). \end{array}\right.$$

(19) $$UACI=\frac{1}{m\times n}\sum_{i=1}^{m}\sum_{j=1}^{n}\frac{\left|C_{1}\left(i,j\right)-C_{2}\left(i,j\right)\right|}{255}\times 100\%$$

where $C_{1}$ is the normal hiding image and $C_{2}$ is the hiding image when the value of one pixel in the original image is changed, M×N is the size of the hiding image. NPCR and UACI under the experimental data are shown in Table 2, indicating that the hiding scheme proposed in this paper can effectively resist differential attacks.

### 5.3. Blind Extraction of Underdetermined Blind-Source Separation

With the above underdetermined blind-source separation, three mixed receiving images are obtained. To separate the mixed receiving images under chaotic hiding, the study applies the method of constructing multi-component complementation discussed in Section 3. The first received signal is selected, and the first intrinsic mode component obtained after the EMD decomposition is used as the fourth received signal. This intrinsic mode function is compensated with the first received signal to obtain the fifth received signal, thereby forming a new received signal vector.

**Figure 9:**
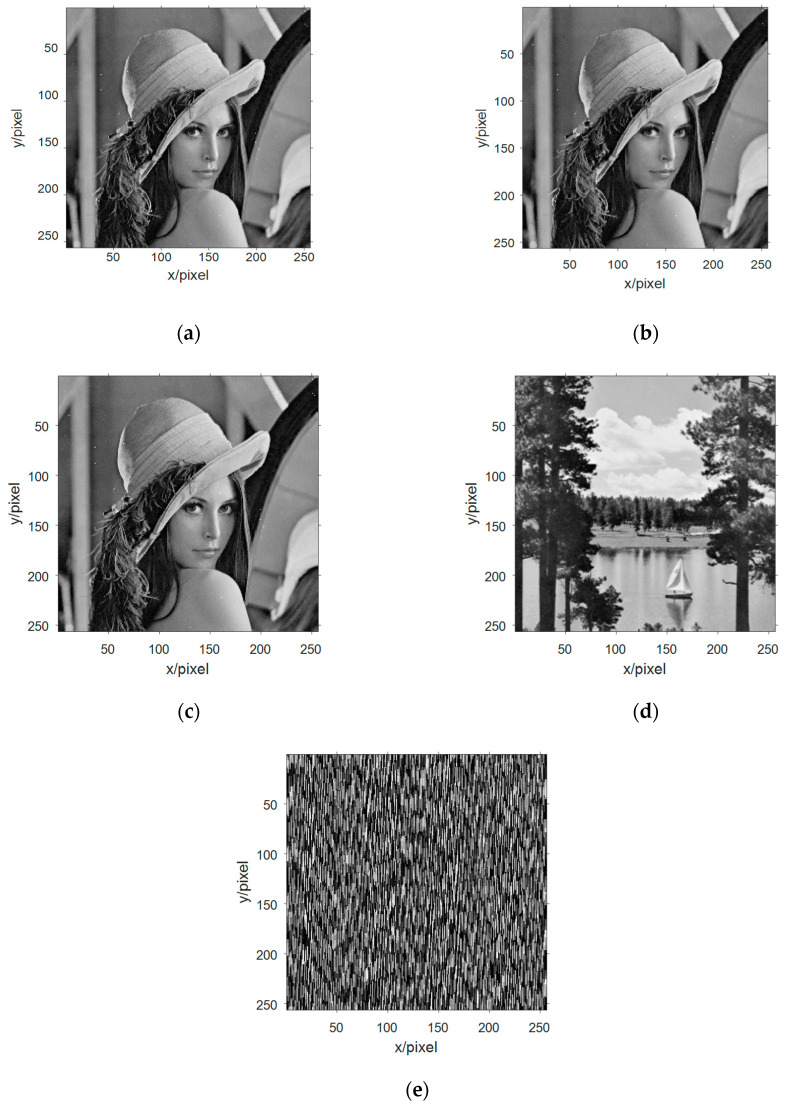
Multi-component complement method first extracts signal image information. (**a**)–(**c**) three effective Lena graphs were extracted blind at the first blind extraction; (**d)** the effective Lake information was extracted blind at the first blind extraction; and (**e**) information of no interest.

The received signal vector is processed by FastICA algorithm to obtain the estimated signal, and the estimated signal value is decimalized and two-dimensional arrayed. The image information obtained after blind separations are shown in Figure 9.

With human subjective visual judgment, it was found that there are four images information similar to the image information in Figure 6, which can be calculated by similarity with similar image information in Figure 6, respectively. Figure 9a and Figure 6a The SSIM is 0.9869, the SSIM in Figure 9b and Figure 6a is 0.9869, the SSIM in Figure 9c and Figure 6a is 0.9869, Figure 9d and Figure 6d SSIM is 0.9990. Based on the evaluation of SSIM, it can be judged that Figure 6a,d have been successfully evaluated. Through calculation, it can be seen that Figure 9a–c are the same image, so the simulation experiment extracts three different image information. The global transmission matrix in this simulation is:

(20) $$G=\left[\begin{array}{ccccc} 0.0488 & 0.0583 & 0.0659 & 0.0649 & 0.0034\\ 0.0273 & 0.0166 & 0.1378 & 0.0113 & 0.9982\\ 0.0545 & 0.1065 & 0.2644 & 0.9943 & 0.0137\\ 0.0217 & 0.0227 & 0.0310 & 0.0049 & 0.0570\\ 0.9967 & 0.9922 & 0.9517 & 0.0984 & 0.0149 \end{array}\right]$$

Observing the matrix, the dominance is 3, corresponding to the three different image information extracted in the simulation. Substituting the values of Figure 9a,d into Equation (11) in turn yields two values. Respectively ${\tilde{w}}_{1}=\left\{\begin{array}{c} 0.2970\\ 0.5159\\ 0.8847 \end{array}\right\}$ and ${\tilde{w}}_{2}=\left\{\begin{array}{c} 0.4801\\ 0.0803\\ 0.6676 \end{array}\right\}$.

**Figure 10:**
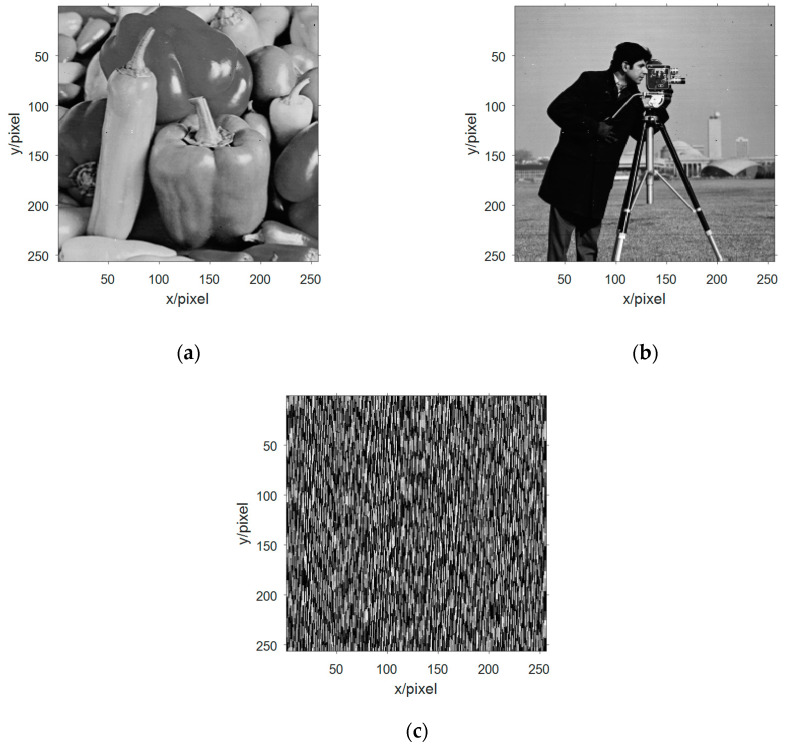
Image information of the second extraction of signal in multicomponent complement method. (**a**) The second blind extraction of the remaining information of valid Peppers image information; (**b**) The second blind extraction of the remaining information of valid Cameraman image information; and (**c**) Information of no interest.

In Equation (10), the original mixed signal value is successively subtracted from the value of the extracted image information in the original mixed receiving signal, and then a new set of mixed signal values can be obtained, and the new mixed signal matrix is subjected to FastICA algorithm blind-source separation. Three-way estimated signal values are obtained, and decimal conversion is performed on it, and the image information shown in Figure 10 is two-dimensionalized.

By comparing the mixed receiving of Figure 10 with the figure, it is found that Figure 10a is an estimate of Figure 6c and Figure 10b is an estimate of Figure 6b. Respectively, the SSIM between them was 0.9943 and 0.9930. The second blind extraction of the multi-component complementary method has effectively extracted the remaining image information.

Shannon’s theory points out that the channel capacity of additive Gaussian white noise channel is the lower limit, which exists widely in all resistive components and has wide spectrum characteristics similar to the chaotic masking signal in this paper. Therefore, in order to verify the effectiveness of the experiment, white Gaussian noise with different intensity is added to the channel to verify the image hiding method of BSS. The results are presented in Table 3.

As apparent from the table, the noise at the source has little effect on the image hiding, and the effect of the image hiding was also reduced as the noise intensity changed.

**Figure 11:**
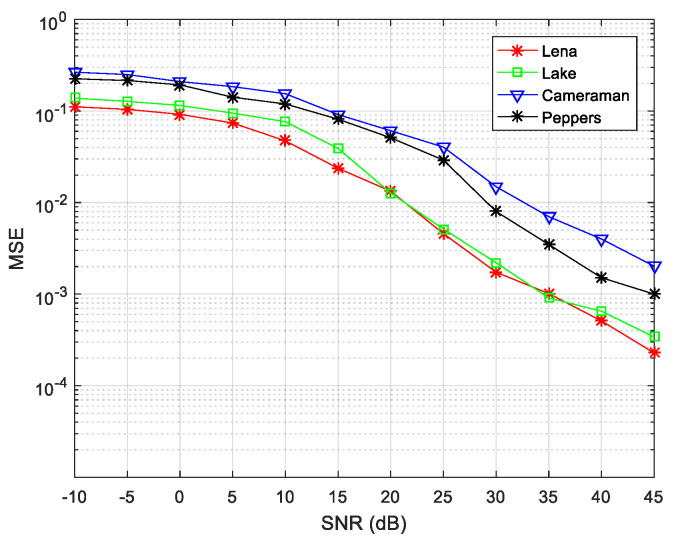
Decryption effects of 5 × 3 signals of source and receiver at different signal-to-noise ratio (SNRs).

The results show that the performance of the underdetermined blind separation algorithm is related to the configuration of the transceiver antenna. We defined the missing degree as:

(21) (N−M)/N

where, N is the number of transmitter signals and M is the number of mixed receiving signals. The above results indicate that the transmitter signal of the simulation experiment was basically restored in the absence of noise. As the above simulation was performed under noiseless conditions, the algorithm performance was examined when the SNR value was changed several times for transmitter and receiver signals of 5×3. The line graph of Figure 11 reveals that the hiding images were all successfully extracted, and the image effect extracted by the first BSS (for Lena and Lake) is better than the second BSS (Cameraman and Peppers).

**Figure 12:**
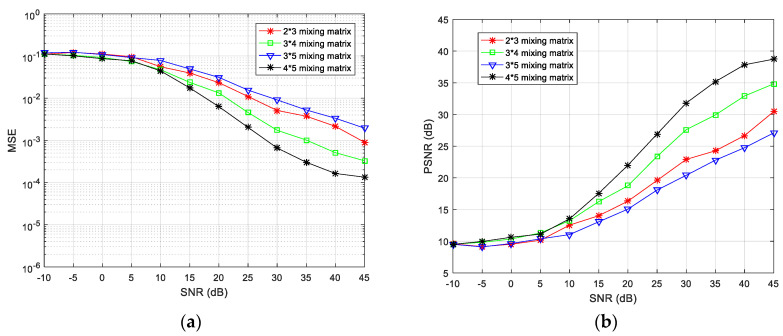
Comparisons of (**a**) mean-square error (MSE) and (**b**) peak signal-to-noise ratio (PSNR) values when the number of source signals and receiving sensors are different.

Finally, to evaluate the overall performance of the proposed algorithm, the number of transmitter and receiver sensors was changed to 2×3,3×4,3×5, and 4×5 simulation experiments were performed. The algorithm encryption matrix was randomly generated and the simulation results at different SNRs are shown in Figure 12.

The simulation results reveal that, as the degree of loss of the receiving antenna element increases, the separation performance gradually decreases. Taking 3×4 and 2×3 antennas as an example, the missing degree of the 3×4 antenna is 1/4, and the missing degree of the 2 × 3 antenna is 1/3, thus, the MSE index is poor for the 2×3 configuration. For the same receiving array, as for the 3×5 and 4×5 configuration schemes, it can also be seen that the MSE index under the 3×5 configuration is poor. In summary, the algorithm proposed in this paper can achieve underdetermined blind extraction under different antenna configurations, and its separation performance is better for a lower degree of missing array elements.

**Figure 13:**
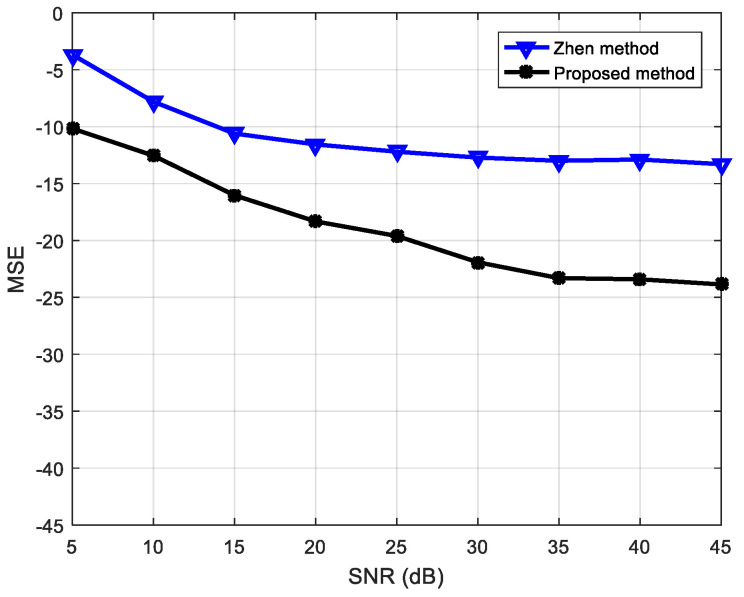
Performance comparison of proposed algorithm and that in [53].

Previously, Zhen [53] analyzed the underdetermined BSS based on a speech signal for a 3×4 antenna configuration. To compare the advantages of the proposed algorithm, the algorithm used in this study was also simulated in the same configuration and compared with the results in [53]. The experiment results are shown in Figure 13. From the MSE values according to the SNR, it is apparent that the algorithm proposed in this paper is superior to that in the literature [53] as regards signal restoration.

In the overall experiment, several aspects should be considered: firstly, the chaotic masking technology is universal for encryption, and the appropriate chaotic system can be selected according to the required situation. Secondly, the core part of this paper is the multi-component complementary method and out-of-order Elimination Algorithm proposed in Section 3. Only through the combination of the two algorithms can we successfully extract the interested sending end signals step-by-step. Finally, our experiment mainly considers the degree of noise and missing degree at the source, which will be further studied in the future.

## 6. Conclusions

In this paper, an image blind extraction algorithm based on an out-of-order elimination algorithm is proposed, and a virtual multicomponent array is constructed to transform the undetermined blind-source separation into a positive definite model. By combining the multi-component complementarity method and an out-of-order elimination algorithm, the image information which is concealed by chaos is extracted successfully. Experiments show that the EMD decomposition technique is used to extract the strongest representation of the received signal, and the virtual receiving signal vector is obtained by the parity cross sequence compensation method in the multi-component complement method. By using the algorithm of out-of-order elimination, the image information can be extracted completely. There are still some deficiencies in this algorithm. In the future, we will analyze the influence of signal source noise, channel noise and receiving sensor noise on the modified algorithm, and verify the effectiveness and universality of this algorithm.

## Figures and Tables

**Table 1:** Image entropy analysis after chaotic hiding.

Image	Observation Signal (a)	Observation Signal (b)	Observation Signal (c)
Lorenz chaos	7.8234	7.8853	7.8156
3D chaos	7.9926	7.9954	7.9927

**Table 2:** Average Number of Pixels Change Rate (NPCR) and the Unified Average Changing Intensity (UACI) values.

	UACI% (Ideal: 33.4635%)			NPCR% (Ideal: 99.6093%)		
	Figure 7a	Figure 7b	Figure 7c	Figure 7a	Figure 7b	Figure 7c
**Lena**	33.3611	33.3864	33.3973	99.5955	99.5672	99.5769
**Lake**	33.3194	33.3504	33.3644	99.5291	99.5122	99.5674
**Peppers**	33.2977	33.3717	33.3935	99.5753	99.5342	99.6062
**Cameraman**	33.3509	33.3509	33.4132	99.4998	99.5552	99.5716

**Table 3:** Relationship between noise intensity and correlation coefficient.

Noise Intensity	0	5	10	15
**Lena**	0.9869	0.9899	0.9845	0.9899
**Lake**	0.9930	0.9942	0.9939	0.9930
**Peppers**	0.9943	0.9930	0.9905	0.9956
**Cameraman**	0.9990	0.9989	0.9969	0.9980
